# The Hospital. Nursing Section

**Published:** 1903-01-10

**Authors:** 


### The Hospital.

### Nursing Section.

Contributions for this Section of "The Hospital" should be addressed to the Editor, "The Hospital" Nursing Section, 28 & 29 Southampton Street, Strand, London, W.C.

No. 850.—Vol. XXXIII. *SATURDAY, JANUARY* 10, 1903.

### Notes on News from the Nursing World.

### THE QUEEN'S MILITARY NURSING SERVICE.

It will be learnt without surprise by those who know the great interest which the Queen takes in the nursing by women of her soldiers, that Her Majesty is expected at an early date to open the new quarters for the nurses of the Herbert Hospital at Woolwich. Accommodation is being provided for an additional staff of forty, who will supersede the orderlies, and the organisation will be on similar lines to those obtaining in large civil hospitals.

### MEMORIAL TO THE PRESIDENT OF THE LOCAL GOVERNMENT BOARD.

The Committee of the Workhouse Infirmary Nursing Association have, we understand, drawn up a memorial which will be forwarded to the President of the Local Government Board, protesting against the creation of "qualified nurses" as proposed by the Departmental Committee on the Nursing of the Sick Poor in Workhouses, viz.: women trained for one year in a small workhouse, and holding a certificate of good conduct and proficiency from the superintendent nurse, chairman of the Board of Guardians, and medical officer. The memorial has been already influentially signed. A copy can be obtained by anyone desiring to sign on application to the Hon. Sydney Holland, The London Hospital, E., or to the Secretary of the Association, at the office, 6 Adam Street, Adelphi, W.C.

### FEMALE NURSES FOR THE AMERICAN NAVY.

It is proposed to provide 50 female nurses for the United States Navy. They are to be taken from the best training schools, and to be subjected to a rigid mental, moral, and physical examination. They will be required to possess sufficient knowledge of medicine to be able to prescribe for emergency patients, and to diagnose cases if necessary. The pay will correspond to that of the army nurse in America.

### THE VALUE OF SICKNESS ASSURANCE.

If any evidence of the value of sickness assurance for nurses were needed, an incident which occurred on Christmas Day supplies an excellent object lesson. A member of the Royal National Pension Fund was pulling a cracker after dinner, and a piece of hot ash flew into her eye. The damage caused was so serious that the victim of the accident is quite incapacitated for work. This unfortunate experience reminds us of the nurse who some years ago called at the office of the Pension Fund in Finsbury Pavement to pay her sickness assurance premium, and when leaving slipped on the stairs and injured her ankle. The injury unfitted her for work, and she obtained sickness allowance forthwith. How liable nurses are to accidents is further shown in the fact that one in every nine of the claims on the Pension Fund for sick pay arises from accidents or operations. Carefulness may do a great deal to prevent disaster; but no class is more exposed to accidents than nurses, and consequently none more urgently needs to have the sickness allowance from the Pension Fund to fall back upon when work is impossible.

### NURSES RETURNING FROM SOUTH AFRICA.

There has been a lull in the arrival of nurses from South Africa. But they have now commenced to return from the concentration camps. On Saturday Civil Nursing Sisters L. Leech, W. Buden, M. McCarthy, L. A. Boret, and A. M. Pease arrived on board the s.s. *Norman.* The day previously the s.s. *Dilwara* reached Southampton, having on board Superintendent A. Garriock, of the Army Nursing Service, and Sister A. Kuys, of the Army Nursing Service Reserve.

### ONE OF THE LADYSMITH HEROINES.

Sister Hill, who has recently been awarded the Royal Red Cross for her services during the South African war, is a native of King's Lynn. At the beginning of the war she was at Capetown, and at once volunteered for the front. She was sent to Ladysmith and arrived there two or three days before the commencement of the now historic siege. After going through all its privations her health broke down when the town was at last relieved, and she came on leave to England. Returning to the Cape she was sent to Pretoria, then in the occupation of Lord Roberts, and nursed British and Boers for some time in the military hospital. She was then transferred to Bloemfontein with a detachment of the South African Constabulary, and was one of the guests of Lord Milner when he recently gave a luncheon to that body.

### THE CHARGE OF BIGOTRY AGAINST AN EDINBURGH MATRON.

From one of the Edinburgh newspapers we learn that Father Power has been prompted by our remarks on the subject of the employment of Roman Catholic nurses in the Edinburgh Royal Infirmary to cite specific instances of alleged intolerance on the part of the lady superintendent. "The organ for nurses," as Father Power describes The Hospital, would have been pleased to afford him the hospitality of its columns, but as he confesses that our demand for evidence in support of his charge has caused him to recognise the need for it, we readily give particulars of his three cases. The first is that of a lady of "purely Scottish extraction" who was asked to call on the matron of the Edinburgh Royal Infirmary, and in the course of the interview was questioned as to the creed she professed. Father Power says that, "On declaring she was a Catholic, Miss Spencer told her, not without some asperity, that she could not be received as a probationer." The second case mentioned is also that of a Scottish girl, who, according to Father Power, "had a precisely similar experience." Lastly, he names the case of an Army Reserve Sister who, "having been summoned before the same tribunal, made the same avowal of her Catholicity, and was instantly informed by Miss Spencer that her application could not be entertained." Father Power adds that these are recent instances of the intolerance of the lady superintendent. It has since been suggested by the Edinburgh paper in which his letter was published that he should appeal to the Board of Management, but his reply is, in effect, that the Board are so completely under the thumb of the lady superintendent that they have lost the power of exercising their natural function of authority. This seems incredible; but if so, behind the Board of Management are the subscribers to the charity. Unless, in acting as she is said to have done, the matron is carrying out the directions of the Board, she should certainly be required to give an explanation in reference to the three cases cited by Father Power, and if the Board should not request such an explanation, the subscribers would have a right to know the reason why.

### PUBLICANS AND DISTRICT NURSING.

It will interest other district nursing societies to learn that the Hendon Association have had a generous contribution to their funds from the publicans of the district. The majority of the licensed victuallers in Hendon have agreed for the future to dispense with the practice of giving Christmas-boxes to their customers and decided instead to forward a donation to the Hendon Sick Nursing Fund. The amount collected was £14 3s. 6d. This is an excellent new departure, and may be commended to the notice of licensed victuallers in other parts of the country. The report of the Hendon Association shows that a great deal of work has been done during the past year. The number of cases attended was 226, and the nurse paid no fewer than 6,270 visits.

### SUPERINTENDENT NURSES AND POOR LAW PENSIONS.

There are two very important points in the report of Mr. Preston Thomas, the inspector for the West of England on Poor Law administration for 1901 and 1902. He is of opinion that thedifficulty of obtaining nurses for workhouse infirmaries is increasingly great, and as to superintendent nurses, candidates with the prescribed qualifications seem often unobtainable. "The Nursing Order of 1897," he continues, "necessarily authorised the appointment, as superintendents, of nurses in office at its date, even if without any technical qualifications for the post, but as such original occupants of the post disappear, qualified successors are vainly sought, and year by year it becomes harder to obtain assistant nurses who have gone through any course of hospital training." Not less striking than this is the candid confession of Mr. Preston Thomas that, "the one point in which the poor-law service might have been expected to afford an attraction to the number of fairly educated women in need of a life's employment, viz., the provision of a pension for old age, has proved a signal failure." Mr. Thomas states that nearly all nurses contract themselves out of the Superannuation Act, "probably because it does not allow them to retire until they have devoted almost a lifetime to the service." He thinks that "if it were possible to introduce a system under which any trained nurse could, after 20 or 25 years' service, claim a pension of £25 or £30 payable by the State, the difficulty experienced by many women of providing for themselves, after middle age, would be to some extent surmounted, and that in such circumstances the service might conceivably attract consideration, especially if means for training them locally were organised."

### TWO QUEEN'S NURSES FOR WILLENHALL.

In the heart of the Black Country, at busy Willenhall, a District Nursing Association has been formed, with the chairman of the Urban District Council for the time being as president. The three resident magistrates have consented to act as vice-presidents, and a strong executive has been appointed to control the work and to assist in obtaining the necessary subscriptions for carrying it on. In order to bring the scheme before the artisan class, eleven representative men belonging to it have very wisely been elected to serve on the management committee. It is hoped that half the sum to meet the expenditure of £180, which will be required to start with two Queen's nurses, will be subscribed by the workmen, who have already manifested considerable interest in the project.

### ADVICE OF A SUPERINTENDENT NURSE TO PROBATIONERS.

At a conversazione held a few days ago in the board-room of the Coventry Workhouse, at which all the members of the Coventry Board of Guardians were present, Miss Whale, the newly appointed superintendent, read a paper primarily intended for probationers. Speaking from her experience as a nurse of twenty years' standing, Miss Whale said that anyone who touched this vocation without a real desire for nursing, a love for it irrespective of the means by which a living can be made, must assuredly tire of it. "I have seen," she continued, "some of the highest in the land take up nursing, but for the most part it has been a castle in the air—a something that might lead to either popularity or an altered condition of life. If you are gifted with the instincts of a kindly, sympathetic nature, you will also find nursing real anxiety, care, and worry. Then if the love and sense of duty to poor suffering humanity are not within you, surely will come disappointment. You will lose the original ardour of your former intentions, and continually waste perhaps the best, vigorous, and most useful period of your life; so that before you finally elect to take up nursing, you must ask yourself the question, and be able conscientiously to answer it: 'Have I this love for ministration to the sick, and do I feel that I can sacrifice my life, with all the disagreeble disappointments of nursing, for the good of my fellow creatures?'"

### HUDDERSFIELD NURSING ASSOCIATION.

The Huddersfield Board of Guardians, we note with pleasure, have not turned a deaf ear to the appeal of the Victoria Nursing Association. Although there was some opposition to the proposal that the board should contribute to the association it was not successful, and the sum of £21 was voted. As evidence of the claims of the association upon the financial help of the board, it was stated that 34 cases had been recently relieved by the former which might have been properly taken to the workhouse. The association is still in want of funds to ensure its permanent existence on a scale commensurate with the need, and some of the rich residents of the prosperous town have thus an admirable opportunity of distinguishing themselves.

### A GUILD FOR POOR LAW NURSES.

It is proposed to form a guild for officers and nurses belonging to the Church of England employed in workhouses and workhouse infirmaries. The idea of the promoters of the movement is that circumstances tend to place such persons in an isolated position without opportunities for religious ministrations. The organisation is to be called "The Guild of the Holy Angels," and the rules to be observed are to be of a simple character. It appears to be the outcome of a suggestion from the Rev. W. H. Frere, Superior of the Community of the Resurrection, who would seem to be somewhat deficient in a sense of humour. We shall watch its development with interest; but, as it is hardly necessary to point out, there are in many cases chapels attached to the workhouses with excellent chaplains.

### CASUAL VISITS BY A QUEEN'S NURSE.

The annual report of the Dunbar, Belhaven, and Spott District Nursing Association, just issued, shows that the work during the year has steadily increased. The Queen's Nurse attended 67 cases, paid 2,059 nursing visits and 147 "casual visits." The record of casual visits is a novelty, but we are sure that they are appreciated when the nurse has leisure to make them, especially on the part of a nurse in reference to whom it is stated that "the committee has heard from time to time many expressions of gratitude from her patients, testifying to the thoroughness of her work and the general esteem in which she is held." Financially, the association has a balance on the right side of no less than £252.

### ENTERTAINMENT AT ST. MARY'S HOSPITAL.

On Friday evening a dramatic entertainment was given by the resident medical officers of St. Mary's Hospital, Paddington, to the nurses and their friends. Each nurse was allowed to ask a friend and every effort was made by the authorities to give these friends a hearty welcome. Two pieces were performed: "My Lord in Livery" and an original farcical comedy in one act entitled "Browne with an E." There was an interval of forty minutes during which a miscellaneous concert was given. After the performance, while the guests were being provided with tea and coffee, the Leoni Ladies' Quintette performed a programme of excellent music.

### ROYAL PORTSMOUTH HOSPITAL.

Gratitude from former patients of a hospital is always acceptable, but perhaps to the nurses it is particularly so at Christmas-time, when there is of necessity extra pressure. At the Royal Portsmouth Hospital the "Prize Ward," as it was called, was beautifully decorated by light festoons of graceful green sprays crossing the ward from side to side, all joining in the centre, united by a huge umbrella. The walls were beautified by Japanese Kakemonor, draped with falling evergreens. The sisters and nurses were not only assisted by the medical staff but by many grateful former patients, who came on Christmas Eve to offer willing service. The children's ward, unfortunately, was closed this year, but those little ones who had been temporarily scattered abroad in other wards had their tree just the same. They also enjoyed the fun of "the light that failed," for the electric light refused to act, and lamps and candles had to be brought to the rescue.

### ENTERTAINMENT TO WALSALL NURSES.

The ladies and gentlemen who form the executive committee of the Walsall Victoria Nursing Institution very kindly provided a thoroughly enjoyable entertainment last week for the lady superintendent and members of the staff. It consisted of a visit to a theatre at Birmingham to see the pantomime, and an excellent high tea at one of the best restaurants, after which the party returned to their home at Walsall for progressive whist and music, the members of the committee waiting upon the nurses and doing all in their power to make the evening a truly happy one. It was not only the entertainment generally, the entire cost of which was borne by the members of the committee, but the thoughtful and kindly action of individual members, which the nurses appreciated—such as the provision of large quantities of flowers for decorating the rooms, prizes, scoring-cards, favours, a great variety of cakes and crackers. A sister of a member of the committee also spent a good deal of time in making up an exquisite spray of choice flowers for every member of the staff.

### WEYMOUTH NURSES.

As usual on Christmas Day all the staff attached to the Trained Nurses' Institute at Weymouth were invited to dinner or supper, and as a large number were nursing in the neighbourhood, a considerable party sat down for each meal in the prettily decorated dining-room. During the day telegrams, cards, and letters were received from former members of the staff, which were very much appreciated. The nurses presented Miss Dibb, the lady superintendent, with a lovely silver cream jug, and to the matron, Miss Cornwall, a bread fork with silver and mother-of-pearl handle.

### SHORT ITEMS.

The annual nurses' conversazione at St. Thomas's Hospital will take place on Friday, January 30th.—Owing to a case of infection, the Christmas entertainment at Paddington Green Children's Hospital has been postponed for at least a month.—The entertainment at St. Mary's Hospital for Sick Children, Plaistow, took place on Monday, December 29th.—In aid of the North Berkshire Nursing Association a special Christmas children's entertainment will be given in the Royal Albert Hall Theatre on Thursday and Friday next week at 3 P.M. The programme is very attractive, and tickets varying in price may be obtained from Mr. Nigel Playfair, 5 Verulam Buildings, Gray's Inn, W.C.

### The Nursing Outlook.

"From magnanimity, all fear above;
From nobler recompense, above applause;
Which owes to man's short outlook all its charm."

### HOSPITAL ECONOMICS AND HIGHER EDUCATION.

Columbia College, New York, has started a one year's course in hospital economics, open only to trained nurses, with the object of preparing nurses to take higher posts in the hospital world, such as superintendents of institutions or lecturers on nursing. Now we are feeling very keenly in England just now a lack of mind on the part of nurses—a need for more capable women of character to take the highest posts in the nursing world and direct the larger branches of what is becoming a most important profession for women. Therefore it is well to study the Columbia course, and see if a similar course carried on over here might prove of value.

There is an entrance examination to the course, at which the examiners have to satisfy themselves that the candidate has completed an approved term in a secondary or normal school, and also in a nurse training school. The time spent in the nursing school must have been two or three years: we note the two years with pleasure in this case, for a woman who can afford to give one year to the theoretical study of hospital economics may well be permitted to do so as a substitute for the third year in the training school, especially as she has to have experience of three or four months of private duty. The fees and cost of living for the eight months' session at Columbia equal £100.

The course includes three lectures by Miss Nutting, of the Johns Hopkins, on the "History of the Hospitals," and Miss Nutting gives a bibliography of books, which includes such works as "Hospitals and Asylums of the World," and the "Review and History of Medicine." Then there are four lectures by Miss Allerton on Hospital Construction, Sanitation and Ventilation; eight lectures by Miss Maud Banfield on Hospital Administration; four lectures by Mrs. Hampton Robb on Training School Administration; and another four on the same subject by Miss M. M. Riddle. The above form the special subjects introduced at the college for this course; but besides the above the students in hospital economics have to study in the Domestic Science School, including household chemistry, dietetics, etc.; on the science side biology, bacteriology, etc.; and on the teachers' side, education, psychology, etc. There is also an opportunity for a little practical work in the laboratories, including culture of bacteria, milk sterilization, and so on; it is therefore obvious that there is a wide field—probably too wide—and plenty to provide eight months of really hard study.

The only course we have in London that in any way touches this is the year's course in hygiene at Bedford College, which is generally taken by women desiring to qualify as health missioners and sanitary inspectors; it was not started for nurses, though a few nurses have taken it up. Bedford College also has a teaching department, and is affiliated with London University; so that by adding the special lectures necessary on hospital economics it could at very little expense, and with very little trouble organise a course somewhat similar to that at Columbia. This is important because it is not likely that many nurses would take up the course at first; not many women with their bread and butter to earn can afford to spend one year of time and £100 in money in securing further qualification than their nursing certificates; it would only be those who were ambitious and clever, and firmly intended to accept nursing as a profession for life who would do so. There are only seven nurses taking the course at Columbia this year. But this difficulty with regard to the special lectures might be met by some of our educational and advanced philanthropists endowing a chair in hospital economics. We are not so proud as the American nurses—who are sadly hampered for funds for their cause just now—and we frankly suggest to any over-burdened millionaire that he should approach the Sectional Committee on Nursing in connection with the Nurses' Club, with the offer of endowment if they will do the organising. It must be remembered that the head of a hospital or infirmary with 600 to 1,000 beds, or the head of a business such as the Nurses' Co-operation, with a turnover of £50,000, or the woman who in the event of a big war would have the organising of the nursing of thousands of wounded soldiers, cannot be too highly trained for her work. So far we have had to rely on the training secured as "sister" or "assistant matron," and very excellent training it has often been; but there is no extra qualification an ambitious nurse can gain—no certificate beyond that secured when she has fulfilled her time in her training school. The mad rush of sentimental damsels into the nursing world has ceased; now the educated woman who turns to nursing turns to it as deliberately as she turns to teaching. And exactly as the teacher who aspires to a headship adds a University degree to her Teacher's Certificate, so a nurse who aspires to a headship should be able to add a University Certificate in general knowledge and the higher branches of hospital administration to that derived from her training school.

### Lectures on Ophthalmic Nursing.

By A. S. COBBLEDICK, M.D., B.S.Lond., Senior Clinical Assistant Royal Eye Hospital, late House-Surgeon and Registrar, Royal Eye Hospital.

### LECTURE I.—INTRODUCTORY.

### ANATOMY OF THE ORBIT.

OPHTHALMIC nursing, like some other special branches of surgical nursing, has been somewhat neglected and treated with but scant courtesy by the trained nurse of three or more years' experience, who, after spending most of her time in nursing general surgical cases, feels that the eye has but few attractions from her point of view.

Not unfrequently she also comes to the conclusion that there is *nothing to do* in nursing eye cases; the nurse who takes up this branch of work very quickly finds that she has made a mistake in forming such an opinion, and, if she is observant and a good nurse, she is forcibly struck by the amount of judgment she must exercise and the manner in which her resources are taxed to the uttermost.

There is no doubt that a three years' general training is a great advantage to a nurse who begins eye work; on the other hand, those who through being under age are not acceptable in a general hospital will, if they enter an eye hospital as probationers, gain much general as well as special knowledge of value to them in their future, whether they take up district, private, or general hospital nursing. To the district nurse a sound knowledge of eye work will render her services doubly valuable to all with whom she comes into professional contact; and it is notorious that in private nursing there is always difficulty in obtaining the services of a skilled "eye" nurse.

Eye nursing requires the qualities which are essential in the making of all good nurses, viz., common sense, cleanliness, ability to attend to minute details and to carry out the orders of the medical attendants, kindness, patience, and a full appreciation of the responsibility of her work.

In the lectures which follow such points will be taken in detail and made as lucid as possible as have an important bearing on points of nursing; other points of essentially medical and surgical interest will be described less fully.

To the nurse who is interested in her work most of these lectures will be full of interest, although sometimes out of her sphere, strictly speaking. There is no reason why an observant and clever nurse should not, when she gets the opportunity and has some guidance, teach herself some points outside her nursing, *e.g.*, if a nurse has at every opportunity taken the advantage of feeling the tension of eyeballs in health and disease, *i.e.*, the degree of softness or hardness of the eyeball, it might be of great help to her and her patients in district nursing, especially in the country.

In the same way, many nurses in taking a pulse do much more than count it; they note whether it is strong and weak, regular or irregular, and also whether it is obliterated by a slight or a considerable pressure of the finger.

In this connection a note of warning must be sounded, viz., that this kind of observation should be secondary, and never interfere with the nursing, which must always be first and foremost; it must likewise never lead her to interfere with the treatment and other details prescribed by the medical attendant; if she cannot subordinate her interest in the medical side of the case to the nursing side, she will do much better to leave nursing alone and, if the circumstances are favourable, commence to work for a medical degree.

It is necessary in all branches of nursing to have a *sound*, if not detailed, knowledge of the anatomy and physiology of the part which is about to be studied.

In studying the anatomy of the orbit and eyeball, a much better and more lasting grasp of the subject will be obtained by having a bony skull and a sheep's or ox's eye in front of you, than by any amount of reading.

*Anatomy.*—The bony orbit. If a human skull is glanced at, several points of importance are at once evident. The shape of the orbital cavity is roughly that of a cone, with the base directed forwards and slightly outwards; this outward direction is partly caused by the obliquity of the outer wall of the orbit; the inner wall passes almost straight backwards and assists in forming the outer boundary of the nasal cavity.

By holding the skull between oneself and a strong light, or by gently tapping the different parts of bone of which the orbit is composed, it is easily demonstrated that the walls of the orbital cavity are in parts thin and in other parts very thick and dense; it is noticeable that the thick portions are so situated as to be a protection to the eyeball from external injury, *e.g.*, examine the orbital margin; the whole margin is composed of dense bone, formed above by the frontal bone, externally by the malar bone, and internally and below by the superior maxillary bone. The ordinary black eye is a good illustration of the value of this bony margin; in nearly all these cases the force of the blow is expended on the orbital margin and not on the eyeball, small blood-vessels are ruptured and effusion of blood takes place into the loose tissues of the eyelids, but the eye is saved.

Above and below, this bony margin is continued backwards as the roof and floor of the orbit respectively: these parts of the orbit are very much thinner; in some skulls the orbital and cranial cavities communicate with each other.

At the anterior and outer part of the roof, you should notice a small hollow only of sufficient size to admit the end of the thumb; this is the hollow for the lachrymal gland. On the inner wall of the orbit, near the margin, is a depression and foramen for the remainder of the lachrymal apparatus, viz., the portion which carries away the tears and small foreign bodies which find their way into the conjunctival sac. The depression is for the lachrymal sac and the foramen is for the passage of the nasal duct into the lower and fore part of the nasal cavity.

The apex of this cone-shaped cavity is formed by two foramina, viz., a large pear-shaped fissure called the sphenoidal fissure, through which certain nerves and blood-vessels pass from the cranial cavity to the orbital contents. Immediately above and to the inner side of this fissure is a smaller circular foramen—the optic foramen—which gives passage to the optic nerve.

Another foramen of importance is the spheno-maxillary, which lies at the junction of the floor with the outer wall of the orbit.

There are two small foramina piercing the orbital margin which are of importance, inasmuch as neuralgia of the nerves they transmit is not uncommon in certain eye affections; one of these, the supra-orbital pierces the frontal bone and transmits the supra-orbital nerve; and the other, the infra-orbital, pierces the superior maxillary bone and transmits the infra-orbital nerve.

The shape of the eyeball does not correspond with that of the orbital cavity; the apex of this cone-shaped cavity is occupied by the optic neve, the origin of some of the muscles of the eyeball, and a well-marked mass of fat in which ramify many nerves and blood-vessels. The eyeball itself lies to the front of the cavity, and is chiefly held in place by its extrinsic muscles. It must be remembered that the orbital cavity is lined throughout by a layer of periosteum, which is a vascular connective tissue supplying the underlying bone with nourishment.

### The Report of the Departmental Committee on Workhouse Nursing.

By Miss WILSON, Treasurer of the Workhouse Infirmary Nursing Association, Member of the Midwives' Board.

ALTHOUGH I am well aware of the value of many of the suggestions in the Report, these have already received such full recognition in THE HOSPITAL that I wish in the present paper to deal with such points only as seem to me to be open to criticism.

In our issue of the 27th the creation of "qualified nurses" was also so fully discussed that it is needless for me to dwell at length on this disastrous and short-sighted plan. Mr. Sydney Holland, in his evidence before the Nursing Committee, said:—"If you will excuse my saying so, doctors do not necessarily know anything about nursing; they are not taught it, and they often do not know anything about it, they learn it in time." Had this statement borne fruit in the minds of the Committee, and had a trained matron been consulted, I venture to think that the proposal of "qualified nurses" would not have been placed before the nursing world. Only a want of appreciation of the work accomplished in nursing reform up till now, and a lack of understanding of the obstacles surmounted, could have made the proposal of such a scheme possible. As the medical and nursing journals seem unanimous on this question, I will pass on to the effect of another recommendation which has not, so far, been noted.

### PROPOSED REDUCTION OF NUMBER OF SUPERINTENDENT NURSES.

Under the Nursing Order of 1897, superintendent nurses are appointed in unions which employ three or more nurses. The committee now recommend that where the ordinary number of occupied sick beds is between 60 and 100, these appointments should in future be optional with boards of guardians. This recommendation, if carried into effect, will probably reduce the number of superintendent nurses appointed from 227 to 136. The proposal is supported in the Report by the opinion of one witness only, that of an inspector who is not a medical man.

What the report gives with one hand it takes away with the other; for while recommending greater powers and responsibilities to superintendent nurses, it is proposed that the actual number of these nurses should be reduced by 91. To show how few workhouses will be affected by the new Order it is noteworthy that in 1901 out of a total of 1,368 appointments as nurses only 81 were superintendent nurses. In future the number will be even less, as the appointment of a superintendent nurse will be optional with Boards of Guardians in the many workhouses which contain between 60 and 100 sick beds. The introduction of a new officer with fresh powers and duties has not always been welcome hitherto, and when such appointments are no longer compulsory in medium-sized workhouses, Guardians will naturally shirk making them.

### POSITION OF NURSES IN WORKHOUSES OF UNDER 60 BEDS.

The position of the nurse in the smaller workhouse remains undefined. Points needing definition are her status in relation to the master and matron, and to the nurses under her, and her own duties; all these remain as they were laid down in 1847! Till such important points are clearly defined friction will continue to be set up, and nurses will not be attracted to workhouse nursing any more in the future than they have been in the past. The report contains, it is true, excellent recommendations, but these when not backed up by an order are practically disregarded by the very Boards who most need to reform their system.

At present many rural Boards of Guardians are unable to realise that nursing has recently made great strides. In relation to this question, the evidence of one of the inspectors, Mr. Preston Thomas, is of interest. On being asked if the guardians and workhouse officials in his district were beginning to recognise nursing as a skilled profession, his reply was:—"I know that one of the guardians said only a month ago, when it was a question of a nurse being appointed who had had no experience, "Well, the less as they knows the better; then us can get 'un into our ways all the quicker." This is not a promising pronouncement at the beginning of the twentieth century, and the inspector would scarcely have quoted the remark if he had not considered it characteristic of the views held by a certain type of guardians.

### THE JOINT APPOINTMENT OF WORKHOUSE MATRON AND TRAINED NURSE.

This recommendation was not supported by the witnesses, whose opinion on nursing matters and whose knowledge of general organisation is of the highest value. For example, Miss Gibson, whose experience in Poor-law nursing is unique, and whose opinion as an administrator cannot be excelled, was, in her evidence, strongly opposed to the suggestion. Sir Henry Robinson said that in Ireland the Local Government Board would only approve of such an appointment "in very small workhouses;" and one inspector stated that he considered the two offices "were incompatible." In a few very small workhouses it might be an advantage for the matron, if trained, to take the general responsibility of the sick wards during the sole nurse's time off duty, but, regarded as a scheme, the plan has many drawbacks. When a workhouse matron acts also as superintendent nurse (par. 72) she will be expected to add to her other duties that of training the class later to be known as "qualified nurses." How can training be undertaken by a woman who has already manifold duties in the workhouse, duties from which no assistant matron, if appointed, can possibly relieve her? Boards of Guardians need to be reminded that sound training does not only mean that a certain number of lectures have been given; it means careful teaching in the sick wards from day to day, and the supervision of inexperienced young women, and practical instruction to them in the care of patients. It is work that cannot be accomplished properly by one who has constant claims on time and attention from other and entirely different departments of the establishment.

Another objection to this recommendation is the danger of infection. How can it be safe or proper for a person who must, in the course of her duty, come in contact with every form of disease and dirt—from the tramp sickening of small-pox to the child developing measles or scarlet fever—to safely undertake maternity cases? Any nursing expert would at once have pointed out to the committee the difficulties that must arise in the attempt to unite such dissimilar and incompatible offices. It is to be hoped that this part of the report will be modified.

### MIDWIFERY QUALIFICATION.

This important subject is not adequately dealt with in the report, except in the case of superintendent nurses (par. 97, 4), in which a midwifery qualification recognised by the Midwives' Board is recommended. This is good so far as it goes, as a tardy recognition of a very important need. But, as I have pointed out, the number of workhouses which supply superintendent nurses is comparatively small, a probable maximum of 136, whereas the total number of workhouses of England and Wales is 671; further, the small rural workhouses, often distant from medical help, are those in which the services of a certifi-

THE REPORT OF THE DEPARTMENTAL COMMITTEE ON WORKHOUSE NURSING—*Continued.*

cated midwife are essential. Par. 63 (1) is not, therefore, reassuring; it runs: "At least one trained nurse, *preferably* qualified in midwifery, should, *if possible*, be resident in every workhouse" (the italics are my own).

Twenty years' experience of the Workhouse Infirmary Nursing Association showed conclusively that for the nurse in small rural workhouses a midwifery certificate was imperatively needed. Every nurse trained by the Association in midwifery was therefore obliged to pass the L.O.S. examination before receiving an appointment.

Grant in Aid of Salaries.

The payment of half the nurses' salaries would be a more satisfactory proposal if it were made clear that the grant will only be given to boards by whom trained nurses are employed, as is now the case in Ireland. If the grant is intended to apply to "qualified nurses" there is some danger of that class being employed more widely than is desirable.

A Comprehensive Order Required.

The absence of any recommendation to fix a minimum proportion between nurses and patients, especially between trained nurses and patients is to be regretted. Many years of advice from the Local Government Board to boards of guardians on this point have failed to produce the changes required to bring the nursing in many workhouses up to an efficient standard. The same remark applies to the *voluntary* adoption by boards of guardians of the recommendations of the Committee respecting the increase of prestige of nurses; of arrangements for the circulation of nurses between town and country infirmaries; of the increase of nurses' comforts; of the choice by nurses of a part at least of their own rations; of the presence of at least one trained nurse in the smallest workhouse; of the direct responsibility of guardians for the proper care of the sick poor; and for the preliminary test-period for probationers. To leave such matters, highly technical and essential as they are, to the well-being of the patients and nurses, to the judgment of boards of guardians, is unwise, and can only lead to mutual misunderstandings. It is regrettable also that the recommendations do not include a national scheme (urged by many of the witnesses) for dealing with the supply of nurses as a whole through some central board with a sufficient supply of training institutions (in no case with less than 200 beds), and with power to continue the inducements for long service contained in pensions, sick benefits, etc.

Finally, experience seems to show that only an Order on the lines of that last issued by the Irish Local Government Board—strong, comprehensive, and definite—can deal effectively with the question of workhouse nursing. Such an order in the nature of things would at first be unpopular with a certain type of guardians, but it is in these cases that recommendations have proved ineffective, and compulsion is essential for reform.

Details of nursing improvements may be left to individual boards, but it is important that the initiative as to principles should be taken by the central authorities.

### The Civil Hospitals and the Army Nursing Service Reserve.

BY THE HON. SYDNEY HOLLAND.

A "Reserve Sister" complains that the civil hospitals boycott the nurses of the Army Nursing Reserve, that the matron of her school advised her not to join, as doing so would be a bar to hospital appointments (the truth of which prophecy she has discovered), and that, if this present attitude is maintained, the Army Nursing Service Reserve must cease to exist or consist entirely of private nurses.

I know nothing of any boycott, but it is a fact that nearly every matron of every London hospital disapproved altogether of the principle of starting a Nursing Reserve for the reasons I will give. But, for the moment, I would like to ask "Reserve Sister" whether, if she were a matron, she would appoint as a sister a woman who, at any moment after the outbreak of war—and we are always at war somewhere—may be called away to go and nurse in a military hospital, say Woolwich or Netley—a woman not selected by the matron herself as specially fit to undertake the nursing of soldiers, but one who had volunteered, perhaps some years before, that if war broke out, she would do this, or would serve abroad. Civil hospitals will gladly undergo great inconvenience to help the country in time of war, but their matrons must keep the management of the nursing in their own hands as far as possible, and settle who can be spared and who cannot, and especially who is fit to go and nurse soldiers, and who is not.

Just what I have described did happen at the Poplar Hospital for Accidents. The sister of the women's and children's wards—trained at "The Royal Free"—whom we could ill spare, was suddenly commanded to go to the Woolwich Hospital to take the place of some army sister who had gone to the war. One cannot blame a matron for not putting women into important posts in their hospitals who owe allegiance to another authority.

"Reserve Sister" is indeed egregiously wrong in making the statement that "all matrons agree that the existence of a reserve of nurses is needed to be drawn on in time of war." On the contrary, saving the matron of the "Royal Free," all those whose opinions I know, and I have been compelled to discuss this question very widely, thought the principle of a reserve of nurses utterly wrong.

"Reserve Sister" writes that "when war breaks out the nursing profession should be ready to meet the need for nurses with a supply of the best and most capable women it can produce." To this all matrons would agree, but it is just this which the existence of the Army Nursing Reserve rendered impossible. Instead of taking the best nurses available in the profession at the moment, the existence of a reserve did compel, and would always compel, the War Office to take the nurses from the reserve, to take in other words a lot of nurses who years before, it might have been ten years before, had said that if war broke out they would be ready to do what every nurse in England is anxious to do, to go and nurse the soldiers. And in the case of the present Army Nursing Reserve, these nurses were not even selected carefully by their several matrons as women specially fitted to undertake this very special work. No, they were self-selected volunteers. Several, whom I happen to know myself, would never have been selected to nurse soldiers if their matron had had the selecting, or had even been privately consulted. (After the war had been waged for some time this was remedied, and matrons were written to privately.) I am well aware that the nurses had to pass before a Board, but provided they had a certificate of adequate technical training and nothing on the certificate against their character, they were accepted.

I must not be taken by this to be writing anything against all the Army Nursing Reserve nurses. Heaven forbid! I know how very good many of them were. I am stating the faults of the system of having a reserve at all, and in so doing, I am compelled to point out where the system failed. I am writing, too, of the reserve as it existed immediately before the war.

Take another instance of the [evils of this scheme of a reserve. An age limit is very necessary to be fixed, above which a nurse should not be allowed to enter the Army Nursing Reserve, but it is positively silly after war is declared, to say to a sister of a large surgical ward: "You are 36, too old to join the Army Nursing Reserve, and so you must not go out and nurse the soldiers." But this was actually said to one of the best and most experienced sisters of the London Hospital, specially selected by the matron, who, however, eventually did get out as the results of very special effort and earned the Red Cross. Even a sister of her standing in the nursing profession had to sign a paper of the Army Nursing Reserve undertaking to go and serve in a military hospital in England if called on to do so! A trained nurse of 40 or 45, if in good health, is perfectly capable of nursing as well as one of 35, and the chances are that by that age she will have added enough experience to her training to render her services invaluable.

A standing reserve of nurses, I repeat, does not give the country what it wants, *i.e.*, the best nurses available at the moment. It only secures to the country the services of nurses who may or may not have been the best when they joined the reserve, but who may have deteriorated since. And, in passing, may I add that there is a danger of nurses engaged solely in private nursing deteriorating. It will be generally granted that they cannot be so up to date as the best of those in the wards of the hospitals. This "Reserve Sister" admits frankly.

When war breaks out the Government can apply to the matrons of all the leading hospitals, and in this way I do not hesitate to say that the Government would be able to secure a practically unlimited number of the best nurses in the profession at the moment when they were needed, all carefully selected by women who knew their special capabilities and suitability. The matrons would not necessarily take them all out of the wards. Every matron knows of many really first-rate private nurses. Nurses working on the co-operation or other similar institutions would apply through the matron of their old school.

It may be urged that it would be a very serious matter to make such heavy demands on the staff of hospitals, and so of course it would be. We have all experienced this during the last year when so many were allowed to leave. But proceedings that may be deplored in ordinary times should, and must, be submitted to when the country is actually engaged in war. In such circumstances everyone has to make the best of abnormal conditions in some form or other. The hospitals in sending forth a number of their best workers would of course pay a certain penalty for doing so. They would be obliged to have more than their normal number of new hands, and submit to the difficulties and inconvenience which such a condition temporarily creates; but then hospitals are the institutions where our nurses are made, so this is only as it should be.

What has happened during the war has caused hospitals the maximum amount of inconvenience with not necessarily the best result to the War Office. After the outbreak of war, individual nurses joined the Army Nursing Reserve, individuals not selected by their matron, but, as I have said, by themselves, and often without any consideration for the hospitals' convenience.

Therefore, while there would be a distinct risk that the hospitals might suffer by workers they would wish to retain insisting on going, the result to the Government would be a satisfactory certainty of a supply of nurses best adapted to the special work.

I have tried to set down why the Army Nursing Reserve was, in the opinion of many, a mistake, but I do not want to be thought to write unkindly of those who had a different opinion, and who gave a vast amount of unselfish work to selecting the nurses and sending them out. I only feel strongly that the whole idea was wrong in principle, and long before this last war when Mr. Brodrick was only Under Secretary of War, and when the Army Nursing Reserve was first formed, I wrote to him, almost in the words I have used above—"If war breaks out and you have to go first to your reserve of nurses, you will not necessarily get the best nurses available at the moment when you want them most."

### Christmas in the Hospitals.

(*Continued from page* 198.)

BY OUR OWN COMMISSIONERS.

### The Hospital for Sick Children.

The little inmates of the Children's Hospital in Great Ormond Street had plenty to amuse them during the Christmas season. On Christmas Eve there was the visit of Father Christmas to fill the stockings; then there were carols from the nurses on Christmas Day and on Tuesday, the 30th, there were Christmas trees. The wards were most tastefully decorated; Alice had chains of fairy lamps, all crimson, suspended across the ceiling; a very pretty plan and a safe one too, as all the chains on which the lamps hung were of metal. On the ward tables were some exquisite flowers—roses and chrysanthemums, pink bavadia and delicate pink and white anemones. The baby's cot had a broad pink bow, the little girls' hair was tied with the same colour, and even the toy goat shared the general distinction and wore a wide scarf for the occasion. White and pale pink were chosen in Alexandra, and here one of the tables was entirely devoted to a fairy doll as big as a child of three or four: she wore gauze wings, carried a glittering fan, and stood on ground covered with snow and sparkling with frost. There was music from a mandoline band; solos on a cleverly constructed square violin with one string, made by the performer; and songs and recitations. As soon as the winter afternoon was darkening the candles on the trees were lighted up, and then busy nurses, helped by visitors, carried round the presents, and the excitement of examining them lasted until the visitors departed and the wonderful day was over.

### The Alexandra Hospital.

For some time past many joys have been in store for the children with hip disease who are fortunate enough to be in the Alexandra Hospital, Queen Square. Just before Christmas a kind Jewish lady gave some Christmas trees, hung with any number of presents. On Christmas Day after a first-rate dinner of turkey and other good things, an entertainment was given by the nurses, who had prepared some very pretty tableaux, consisting of scenes from the children's favourites—"Bo-Peep," etc.—and a novel contrast called "Past and Present," This was a "Gamp," stout, beery, and incapable, and a nice, trim, well-set-up modern nurse in uniform—everything that Mrs. Gamp was not, and all that her modern successor is or should be.

### CHRISTMAS IN THE HOSPITALS—*Continued.*

### East London Hospital for Children.

Good taste in ward decorations is always a marked feature at Shadwell, and this Christmas was no exception to the rule. Graceful trailing ivy and smilax, as well as roses and chrysanthemums, were arranged on ward tables, and windows and doorways were outlined with green tracery. What the children always call turkey and plum pudding formed the dinner on Christmas Day, and as a great treat the nurses had tea with the children. Father Christmas went his rounds distributing toys, and on New Year's Eve the event of the season took place in the out-patients' department. There were two large trees with a wealth of toys, and "Daddy Christmas" was greeted with rapture by little patients on mattresses, beds, or in the arms of kindly visitors. The time-honoured Punch was superseded this year by a very clever conjuring and ventriloquial entertainment.

### North-Eastern Hospital for Children.

Christmas at the North-Eastern Hospital for Children, Hackney Road, is being celebrated in homely and hearty fashion. On Christmas Eve the house physician, attired as Father Christmas, accompanied by the resident medical officer, dressed in spangled muslin to represent Jack Frost, went the round of the wards inquiring whether the children had been good and whether all deserved presents. The answer was, of course, in the affirmative in every case, and on Christmas Day, Father Christmas, with the same retainer, again visited every bed, this time loaded with large sacks full of toys which he impartially distributed amongst the little patients. Christmas Day was commenced with carol singing by the children and nurses, and at midday the three resident doctors took charge of the two wards and served the dinners to the children, much to the latter's delight. This is done in order to give the nurses an opportunity of all meeting together at their Christmas dinner table. On Boxing Day Punch and Judy visited each ward, and subsequently each little guest then received an orange, some cakes, and a toy, and the amusement consisted in a cinematograph, provided and worked free of charge by a friend of the hospital.

### The National Hospital for the Paralysed.

A very happy Christmas Day was spent here. There were dinners and ward teas, and the nurses worked hard to ensure the patients under their care having a good time. The decorations, though not elaborate, were pretty and effective, and the fact that the patients themselves helped to prepare them added greatly to their value. In one ward the colours chosen were scarlet and white, and a very delicate girl made some charming paper roses for the lamp shades, while the patient in the next bed strung many snowflakes of cotton wool on white thread, so that there seemed to be a perpetual storm falling from brackets and lamps, and down the looking-glass on the wall of the day room, while red paper chains formed a maypole from the centre of the ward. "David" was in white and green, with twisted chains of tissue paper along the walls, and evergreen and flowers in the middle, with green and white lamp shades. On the Tuesday following Christmas Day entertainments were given in two wards.

### The London Temperance.

Each patient at the London Temperance Hospital was allowed to invite a friend to tea on Christmas Day, and another at six o'clock, when an impromptu entertainment was given in the large ward, which is empty for lack of funds. The ward was decorated with festoons of evergreen suspended across the ceiling and large Chinese lanterns, some of which shaded the electric lights. A feature of the evening was a cats' chorus, in which only the faces of the cats were visible. The annual entertainment was on December 30th, when a large Christmas tree from Scotland was the chief centre of interest. The ward was well filled by patients, nurses, and visitors. A figure of Father Christmas stood in the centre of the room. The chairman of the hospital (Mr. Alderman Strong) presided, and songs and recitations were given by Messrs. J., C., and A. Horncastle (brothers of one of the nurses), Mr. Morrison, Miss and Sister Packe, Nurses Rose, Janet, and Bertha, Miss Lambert, and others. The great event of the evening, however, was the Toy Symphony, *Schlitten-partie*, by the members of the nursing staff, Miss M. Packe, and Mr. A. Horncastle. This was a very pretty performance, and was applauded to the echo. The conductor was Miss Collins.

### The Hospital for Consumption, Brompton.

The Christmas entertainment for the patients took place on New Year's Day, when a splendid tree was erected on the stage of the concert room, and Dr. Ascherson appeared as Father Christmas to dispense the presents. His speech, which concluded with some original verses, was rich in topical allusions, and was received with a good deal of applause and laughter. A crowd of residents and nurses helped to distribute the gifts, and the evening concluded with an excellent conjuring performance by Mr. Charles Bertram. There were, as usual, a number of visitors.

### The Homœopathic.

There were trees in the wards of the Homœopathic Hospital on Christmas Eve; on Christmas Day the nurses sang carols, and there was the usual old English fare for the patients. Boxing Day was devoted to the out-patients: tea and an entertainment and parcels containing things useful and ornamental were the order of the day; there was warm clothing for those in need of it and there were toys for the children. The magnificent tree which had been the delight of the children in Barton Ward for days past had its fruit plucked on New Year's Eve, when visitors came, and there was music and singing. By about six o'clock all festivities were over, and the wards returned to their usual condition of quietness. The decorations, consisting chiefly of evergreens and flowers, were of a kind quickly put up and easily removed.

### The City of London Hospital.

Christmas Day was a grand success at the City of London Hospital, and much praise is due to Miss Fox, the matron, for her untiring efforts in organising arrangements for the pleasure and enjoyment of both patients and nurses. At 6 A.M. carols were sung outside the ward doors, and were much appreciated by the patients, who were already elated at having each received a parcel containing useful garments. The wards were tastefully decorated, and many willing hands made preparations for the midday meal. In the afternoon the patients were entertained with music, vocal and instrumental, and games. The evening was given up to the entertainment of the nurses; the scene was one of true Christmas fun, the matron and her staff entering into the proceedings with energy and goodwill.

### The Station Hospital, Portsmouth.

Christmas Day was observed in a quiet manner in the wards of the Station Hospital at Portsmouth, little beyond a few evergreens about, and sundry additions of seasonable fare to the ordinary diets marking the occasion. On boxing day, however, the patients had a very good time, all the convalescents, to the number of about ninety, sitting down at 4.30 to a bountiful tea arranged by the sisters on tables decorated with plants and flowers, in an empty ward. After tea the men were assembled in another room for a concert. The decorations of this room deserve a word all to themselves, being most elaborate. The ceiling was entirely covered by chains of coloured paper from which Chinese lanterns were suspended. The walls were ornamented with beautifully-made mottoes and devices. None of the famous leaders in the late campaign were forgotten, one corner of the room being specially noticeable, as it was devoted to the names of the five R.A.M.C. officers who had won the V.C. in South Africa. Other devices bore legends of good will to the R.A.M.C. officers, nursing sisters, and the sergeant-major of the hospital. Facing this shield at the other end of the room a good-sized stage was arranged, prettily draped with bunting and strings of fairy lamps. The programme was arranged by Superintendent Sister Payne, and consisted of a capital selection of instrumental and vocal music and recitations. Captain N. Thurston, R.A.M.C., made a most efficient chairman, and Miss Weston, matron of the Garrison Female Hospital was equally good as accompanist. The loyal strains of the National Anthem ended the programme, and after giving three cheers for Sister Payne the patients returned to their wards.

### The Royal Isle of Wight County Hospital.

The decorations at the Royal Isle of Wight County Hospital were very original this year. The children's wing was exceedingly pretty. In the covered way joining the wing to the hospital was a life-size Father Christmas standing in a terrific snow storm, with a huge barrow full of beautiful toys, and on entering the spacious corridor leading to the ward there was found a fascinating scene of the "Babes in the Wood." A little in the background was the horrible face of the ugly uncle peering down on the two sleeping babes. Still further along the corridor was a very pretty musical fountain. On all the window ledges were some of the nursery rhymes, "King in the Counting House," "Queen in the Parlour," "The Maid in the Garden," "Boy Blue," and others. On Christmas Day everyone who was able partook of turkey and plum pudding, and great was the amusement of those who discovered a threepenny piece, a ring, a button, or a thimble. During the afternoon a huge bran-tub full of presents on the men's floor and a cracker large enough to hold 22 fair-sized presents on the women's floor were distributed.

### Friedenheim Hospital.

For the Christmas festival at Friedenheim, turkeys and plum puddings, toys and crackers, and flowers and holly for decorations, poured in. Many little family groups gathered round the beds in the prettily decorated wards on Christmas Day, and a sensation was caused by the arrival, not of Father Christmas, but of a lady in full Chinese dress. She visited each ward in succession, and distributed toys and sweets from well-filled baskets carried by her attendants. Although she spoke only Chinese, she was quickly recognised by all the patients, and especially by the children, as Nurse Ford, late of the China Inland Mission; but the recognition only added to the pleasure of her visit. There was a small but very pretty Christmas tree in the children's ward. A number of Miss Davidson's friends were present during the afternoon, and carols and music were given at intervals.

### East-end Mothers' Home.

The Christmas festivities at the East-end Mothers' Home were kept exclusively for the in-patients. Owing to the liberality of various members of the committee and other kind friends each woman received a most useful present of woollen clothing for herself and baby, as well as each husband and eldest child at home having a small gift. The women thoroughly enjoyed an excellent dinner of turkey—a "real treat"—and custard puddings. The husbands were invited to tea and to spend the evening with their wives. The singing of carols by the nurses and other friends helped to pass the time pleasantly. The wards were prettily decorated with holly and flowers, interspersed with fairy lights.

### Everybody's Opinion.

**[Correspondence on all subjects is invited, but we cannot in any way be responsible for the opinions expressed by our correspondents. No communication can be entertained if the name and address of the correspondent are not given as a guarantee of good faith, but not necessarily for publication. All correspondents should write on one side of the paper only.]**

### OUR CHRISTMAS DISTRIBUTION.

"The Matron of the Great Northern Central Hospital, Holloway Road, N.," writes: Please accept the best thanks of the patients in the Great Northern Central Hospital for the nice things you so kindly sent to help make their Christmas bright and happy. They had a very nice Christmas, and feel very grateful to all those who kindly thought of them. We are grateful to those who gave us the means of making Christmas nice for them.

### AN APPOINTMENT.

"Miss Jessie Coutts" writes from East Poorhouse Hospital, Dundee: Will you please allow me to state that I was trained at the Aberdeen Royal Infirmary and Glasgow Maternity Hospital, as well as at the City Hospital, Aberdeen.

[The appointment was inserted precisely as sent to us.—Ed. The Hospital.]

### OPPRESSED BY POVERTY.

"The Rev. Francis W. Fisher, St. Jude's Vicarage, Peckham, S.E.," writes: In these parts we are oppressed by poverty and neglect and just do what we can to help one another. If you could send some good churchwoman to read to and comfort our aged and neglected I should be grateful. I don't want her to give them any help in money.

If any of our readers is moved to render this measure of personal service perhaps she will kindly write to Mr. Fisher, giving full particulars and asking for an appointment. We should be glad to have a report of her experience in the work if she undertakes it.

### THE JUNIUS S. MORGAN BENEVOLENT FUND.

"A policyholder" writes: I am deeply concerned, as one of the first members of the R. N. P. Fund for Nurses, to see in a recent number of The Hospital that the benevolent branch is not as well supported as it should be. Doubtless this is partly due to the busy lives nurses lead with little time to devote to their own business. May I suggest that with the New Year all nurses, members of the fund, should, if they are able, agree to give 1s. a quarter towards the benevolent branch? This would be of great use to that branch, and an easy way of helping it. I have done this for some years, and hope to do so always when I am earning a salary, as a slight recognition of the blessing the fund is to the members.

### THE STERILISATION OF UNDERCLOTHING.

"Policy No. 8706" writes: In The Hospital of December 20th the evil consequences of not thoroughly sterilising underclothing is quoted from the *Lyons Medical.* The question is asked: "How, then, about woollen materials which, lest they should shrink, are merely washed in tepid water?" With regard to Welsh flannel I think it may be of interest to others to know that Welsh flannel cannot be washed in too hot water, and that it is the better for being boiled. I have skirts in wear now that have been in almost constant wear for the last nine or ten years. They have been boiled many times; they are not shrunk, and are as good a colour as when new. It is true that though white Welsh flannel does not shrink after boiling it turns yellow, but I find the plain grey everything that one could wish. I may say I have steeped my skirts in strong disinfectant and boiled them after, but in that case I had to wash and boil them a second time to clear them. No soap must be put into the boiler. Plenty of soap is used to wash the flannel so as to make a good soapy lather, but after washing the flannels must be plunged into the boiling clean water and kept boiling for five, 10, or 15 minutes as is found necessary.

THE NURSES' CO-OPERATION.

MISS THERESA H. THORP writes under date January 5th: In your issue of January 3rd I notice you mention the "present scandal in the greatest society of private nurses—the Nurses' Co-operation." As members of the staff of that society several of us would like to know to what you allude. At the present time our affairs are working smoothly, and we would prefer not to have a wrong impression given to the general public. It can but do our society harm. I should be obliged if you would publish this.

[Our columns have contained full particulars of the mismanagement of the Nurses' Co-operation referred to. We refer our correspondent to the articles in THE HOSPITAL of March 16th, May 11th and 18th, and June 22nd, 1901, and of October 25th, 1902.—ED. THE HOSPITAL.]

### A Plasmon Demonstration.

BY A NURSE.

I HAD seen from an advertisement in THE HOSPITAL that any day nurses would be welcomed at 56 Duke Street, Grosvenor Square, London, W., to see practical demonstrations made with Plasmon essence. Being anxious to know more about this wonderful new food, I went, and was much interested in what I saw. I think most nurses would be the same. Of course all know what Plasmon is—the proteid of milk—but perhaps all may not know that it is called Plasmon because it is "that which makes" tissue and muscle. The first thing shown me was the "stock," which at first sight looked almost like chicken jelly. This can be kept in a cool place, for a couple of days and added to soups, vegetables, blanc-manges, infant's food, etc., greatly increasing their nutritive value. If preferred it may be warmed and taken alone, sweetened or plain. Next I saw how to make this jelly properly, using just the right proportions—three teaspoonfuls of the powder to half a pint of tepid water—and was also shown that by adding a small quantity of salt the mixture could be turned back into what had every appearance of being milk. Next I had a cup of Plasmon cocoa, and with a little sugar it was delicious—not thick and starchy, but very fine, leaving no appreciable sediment in the cup. It is strained in a dry state, I understand, through silk. With the addition of cream, however, it was, as old Soyer used to say of his dishes, "fit for beings better than men." The cream was made simply by beating up some of the Plasmon stock with an egg-whisk, and this cream, when flavoured, say, with vanilla makes a delightful addition to puddings or fruit. It may be used for meringues, as it tastes just like Devonshire cream without the fat.

The value of Plasmon has been highly extolled by the medical Press as well as by several eminent doctors. It appears to be especially useful in cases of weak digestion, after gastric illness, and for babies who cannot assimilate milk. As a contrast to these little patients no less a personage than Mr. Sandow is enthusiastic about its nutritive and staying powers, and says that when using it he never experiences any sense of fatigue after his work is over, on the contrary feeling as fresh as when he first began. Most nurses would like to be able to say the same. Wherefore try Plasmon. There are various other forms of Plasmon—chocolate, beef plasmon, and bread and biscuits. When I came away I was given a cookery book containing a large number of recipes for cooking Plasmon, and I believe that any nurse who goes to see the demonstration may have one if she wishes.

### Appointments.

[No charge is made for announcements under this head, and we are always glad to receive, and publish, appointments. But it is essential that in all cases the school of training should be given.]

CHESTERFIELD AND NORTH DERBYSHIRE HOSPITAL.—Miss Beatrice Platt has been appointed sister of women's surgical ward. She was trained at the North Staffordshire Infirmary and Eye Hospital, and has since been charge nurse at the Jubilee Hospital, Earl's Court, London.

HUDDERSFIELD INFIRMARY.—Miss Emily Barry has been appointed matron. She was trained at the Wirral Children's Hospital, Birkenhead, and Westminster Hospital. She has since been sister of the men's corridor, Hospital for Diseases of the Chest, Victoria Park, assistant matron in the same hospital, and superintendent of the Nurses' Home attached to Queen Charlotte's Lying-in Hospital, Marylebone, London.

SOUTHAMPTON ISOLATION HOSPITAL.—Miss Sarah Dalton has been appointed lady matron. She was trained at the Chester General Infirmary and the Monsall Hospital, Manchester. She has since been matron at Mitcham Isolation Hospital and matron at St. Alban's Hospital for Infectious Cases.

### Presentations.

MILNTHORPE AND STORTH NURSING ASSOCIATION.—Miss M. J. Shepley, who has just left Milnthorpe for Belfast, was presented prior to her departure with a silver afternoon tea set, silver-backed toilet glass, and silver photograph frame by her numerous friends and patients in recognition of her unwearied services as district nurse during a period of five years.

PAIGNTON NURSING INSTITUTION, SOUTH DEVON.—On Christmas morning the lady superintendent of Paignton Nursing Institution, Miss Ethel Gray, was presented with a very handsome embossed silver card case from her staff of nurses. In the evening the nurses in the home took dinner with Miss Gray and spent a very pleasant time.

### "The Hospital" Convalescent Fund.

THE Hon. Secretary begs to acknowledge with thanks the receipt of 1s. from Nurse Burgess, and of 2s. 6d. from "Alison," who is also thanked for her kind promise of an annual subscription.

### TRAVEL NOTES AND QUERIES.

ST. JEAN-DE-LUZ FOR SIX WEEKS (Espagne).—I think you have decided wisely. Write to Mme. Dumas, Hôtel de la Poste, and ask if she will take you for a few weeks at 6.50 or 7 francs. The Hôtel d'Angleterre will also take you for about the same price; but go to the Poste if you can—the Dumas are such nice people. It is not warm; take the same clothes you would wear in England. No evening dress needed. This hotel does most of the posting business, and carriages are good and cheap. When there, explain that you will often be out to midday meal, and ask if you may take some bread-and-butter and cheese out with you then. You can make the ascent of "La Rhune" and the "Trois Couronnes" from thence, and visit San Sebastian, Passajes, and Fuentarabia. In crossing the frontier at Irun mind you do not find yourselves cunningly made to carry contraband articles by astute Spanish peasants. It is no uncommon thing to find your fellow travellers, after passing the station, divesting themselves of rolls of clothing, and from stout and comfortable individuals all at once becoming lean and poor-looking. When crossing to Fuentarabia ask on the little quay for Eli. Get your tickets through Gaze and Sons, 53 Queen Victoria Street. Cheapest route Newhaven and Dieppe. Approximate fare, second return for 45 days, £6 12s. Look out for articles of mine about the end of February on these places. They will be useful to you. Tell me if I can help you further.

### Echoes from the Outside World.

### The Emperor of India.

Amidst scenes of wondrous Oriental magnificence King Edward was proclaimed Emperor of India in State Durbar at Delhi on January 1st. The Durbar amphitheatre was horseshoe in shape, and accommodated 15,000 spectators. Under the Royal flagstaff in the centre of the arena the guard of honour was furnished by the Gordon Highlanders, and altogether 40,000 men—horse, foot and artillery—were drawn up in panoplied array under the supreme command of Lord Kitchener. The arrival of the veterans of the Mutiny caused the greatest excitement amongst troops and spectators alike. They mustered about 600 strong, Europeans, Eurasians and natives. Of the first, some were in plain mufti, others in uniforms long since discarded, and tarnished and faded in the course of years, and others still wearing the uniforms of their civil or military employment. The natives mostly belonged to the humbler classes, and their long flowing garments were plain and unadorned. One native veteran was said to be 100 years old. The Duke and Duchess of Connaught arrived just before the Viceroy and Lady Curzon, who were in a State carriage, drawn by four splendid horses, with outriders in scarlet and gold. The ceremony of the proclamation was most imposing, accompanied by the roll of drums, the flourish of silver trumpets, and the shouts of thousands of voices. During his speech Lord Curzon read the King-Emperor's message to his people, which was received with loud cheers, especially the allusion to the promised visit of the Prince of Wales. Later the ruling chiefs, 98 in number, did homage, one being a lady, the Begum of Bhopal, who wore a crown above her blue purdah, and one an infant carried in his mother's arms. The next evening a gorgeous display of fireworks delighted the natives, and the set piece, showing portraits of the King-Emperor and other exalted personages, elicited much applause. A State Service took place on Sunday; and on Tuesday the Sikh chieftains marked their sense of the significance of the Durbar and their loyalty to the King-Emperor of India by a solemn act of worship at the shrine of Teg Bahadur, their ninth Guru, who was put to death after foretelling the advent of the British rule.

### Mr. Chamberlain in the Transvaal.

Pursuing his tour in South Africa, Mr. Chamberlain, after a visit to Spion Kop and Ladysmith, where he made an important speech on the railway question as it affects Natal and South Africa generally, left for the Transvaal on Saturday. On the way thither he received and acknowledged addresses from the municipalities of Dundee and Newcastle. At Charlestown, the border town, he was met by Lord Milner and Sir A. Lawley, who joined the train. The proceedings at Volksrust, in the Transvaal, were marred by faulty arrangements, and though a large concourse assembled outside the station, only twelve people were admitted to the platform to hear the address read and the reply of the Colonial Secretary. At Standerton Mr. Chamberlain and Lord Milner inspected the guard of honour provided by the Scottish Rifles, and at Heidelberg Mr. Chamberlain delivered a short speech. Pretoria was not reached until just before midnight. On Monday a reception in honour of the Colonial Secretary and his wife was given by the Lieutenant-Governor of the Transvaal. Lord Milner was present, and the attendance included Louis Botha, Delarey, Cronje, and other prominent Boers. Four addresses were presented by the Town Council, the Chamber of Commerce, and representatives of the Jewish community and of Asiatics to Mr. Chamberlain, who, in acknowledging it, said that the success of his mission was dependent on the co-operation of the people of South Africa.

### Woman's Work at the St. Louis Exhibition.

At the exhibition to take place in St. Louis in 1904 there is to be no separate exhibition of woman's work, nor any separate women's building. For the first time women exhibitors will be placed on the same footing as men both in theory and in fact. Their work will be shown side by side, subjected to the same conditions and tests, measured by the same standards, judged by the same juries, and awarded the same honours. This new arrangement will simplify the classification of exhibits, not only for exhibitors and jurors, but also for visitors, who have necessarily hitherto found it very difficult to make any genuine comparison between work exhibited in the men's section and that shown in the women's department. One proviso has been laid down with regard to the awards. When any exhibit has been produced either wholly or partly by women, a member of the jury examining will be appointed by the Board of Women Managers, a body created, and its powers defined, by Act of Congress.

### Old Masters at the Academy.

The two great features of the winter exhibition in Piccadilly are the works of Albert Cuyp and some examples of the British landscape school going back as far as Constable. So varied is the collection of canvases got together that it may be truly said there is something to suit the taste of every lover of art. There are two or three Gainsboroughs, notably "The Fallen Tree," "Going to Market," and "Landscape," first-rate specimens of the painter's style. Richard Wilson, who by many is looked upon as the founder of the English school, has seven pictures in all, one large "Lake Scene" being a masterpiece. Another in quite a different style is "Rome from Monte Mario." Two of Constable's most famous pictures are shown, "The Leaping Horse" and "The Lock," the latter the artist's diploma picture. "Salisbury from the Bishop's Gardens," is one of the many studies of which one finished example is at South Kensington, and a second has been more than once exhibited. Another picture, originally known as "Salisbury Cathedral from the Meadows," now shown under the title of "The Rainbow," is undoubtedly one of the finest pictures Constable ever painted. Of the five specimens by Sir Joshua Reynolds "Mrs. Pelham" feeding pigeons will attract most attention, and of the six Turners "Harlech" should perhaps rank first.

### Holiday Entertainments.

"Mother Goose" at Drury Lane Theatre this year is a pantomime of the usual gorgeous type. Mr. Dan Leno takes the part of the title rôle and Mr. Herbert Campbell is his chubby, mischievous son, Jack. The principal girl, Miss Madge Lessing, acts well, and has a specially pretty scene where she sings about the witch behind the moon, accompanied by a chorus of children with lamps. "Gooseland" is a most amusing place, where there are schoolboy-geese, horse-geese, soldier-geese, and policemen-geese, and the Land of Heartsease is a veritable dream of fairyland in every shade of colour which the beautiful flower can assume. "Buffalo Bill" at Olympia seems likely to repeat the success which he obtained in London more than a decade ago. He has no longer locks of raven hue, his curls having become decidedly grey, but he sits as firmly in his saddle as of yore, and his eyesight is as keen as ever, for his shooting is superb. The strength of his voice will probably astonish those who have never before heard Colonel Cody speak. Cowboys, Cossacks, Mexicans, Arabs, Gauchos, and Indians all take part in the entertainment, and the riding is of a very high order.

### For Reading to the Sick.

THE EPIPHANY.

WHERE proud Euphrates day by day
Winds through the plain, or sleeping lies,
The watching Magi nightly pray,
And seek the future's hidden way
From planet-lighted skies.

Lo! as they gaze with deep intent,
A Star more brilliant than the rest,
The Herald of some great Event,
Moves through the gilded firmament
Onward towards the west.

Where'er it leads, that fiery Light
Unhidden by the blaze of day,
And marking with intenser might
The darkness of the deeper night
They follow on the way.

With morning's blush, when sunsets fade,
On over rock and steep and wild,
By Palm and Cedar tree and shade,
Till in the homely Manger laid
They find the Royal Child.

*Kittermaster.*

Viewed in one light Epiphany seems well-nigh the most joyous of Christian feasts: in the sense, that is, of its having no gloomy side.

For before this festival our dearest Saviour has already made that incalculable descent which His Incarnation involved.

He has been overlooked by the first instalment of His own who received Him not: witness, His birth in a stable.

He has deigned to shed the first drops of His pure blood, thereby "fulfilling all righteousness" in the rite of Circumcision.

All those humiliations are over.

Even Herod and his troubled Jerusalem have been weighed, found wanting, and for the moment left behind.

And now the wise men arrive with gifts, worship, love; their gifts sanctified by worship, their worship vivified by love.

Did those three alone see the star? Presumably not. Did others seeing arise? We read of none such.

Faith and good will made all the difference between seer and seer.

As then, so now.

The starry heavens are so far like their (and our) Maker, that they answer and instruct each man according to his honest intention, his tolerated stumbling-block, his bosom-idol, as the case may be.

To some they say nothing.

Some they address through the intellect exclusively.

While to Magi (that is, to Wise Men) they declare the Glory of God, and show His handiwork.—*C. Rosetti.*

So with reverent awe we greet Thee,
Name most blessed to our sight,
Holy Jesu, we entreat Thee
In our hearts Thy Name to write,
Till that face to face we meet Thee,
Gathered to Thy Saints in light.

*Latin Hymn.*

### Notes and Queries.

**The Editor is always willing to answer in this column, without any fee, all reasonable questions, as soon as possible.**

**But the following rules must be carefully observed:—**

**1. Every communication must be accompanied by the name and address of the writer.**

**2. The question must always bear upon nursing, directly or indirectly.**

**If an answer is required by letter a fee of half-a-crown must be enclosed with the note containing the inquiry, and we cannot undertake to forward letters addressed to correspondents making inquiries. It is therefore requested that our readers will not enclose either a stamp or a stamped envelope.**

*South Africa.*

(109) Will you kindly give me the addresses of asylums in South Africa; and also the salaries of male attendants?—*Male Nurse.*

Write and ask Dr. W. J. Dodds, Valkenberg Hospital for the Insane, Mowbray, Capetown.

*India.*

(110) I shall be glad if you will give me particulars of the Up-Country Nursing—and any other nursing—Association of India through which a fully-trained nurse could get an appointment, either for hospital or private work?—*C. F. S.*

The Secretary, Mrs. Sheppard, 10 Chester Place, Regent's Park, N.W., will give you particulars of the Up-Country Nursing Association, and the Lady Superintendent, the Ceylon Nurses' Association, Colombo, could supply those respecting nursing on the island.

*Midwifery.*

(111) Is it possible to learn midwifery without paying a premium, and without hospital training? I would give my services.—*A. D.*

How can you learn a difficult thing and give your services at the same time? A midwife can earn from £60 to £100 a year, and yet people think that they can be taught how to do so for nothing.

Will you kindly tell me if there is a lying-in hospital at Liverpool where midwifery pupils are trained, and where a certificate and board-residence could be obtained?—*Nurse M.*

The Liverpool Ladies' Charity and Lying-in Hospital, Brownlow Hill. The fee, including board-residence for three months, is £18 18s. and £1 1s. for laundry.

*Situation.*

(112) My sister would like to get a situation as nurse-companion to a lady or to an invalid child, will you kindly tell me if there is any agency through which she can do so? I have been told that it is of no use to advertise, but only to answer advertisements.—*A District Nurse.*

Certainly advertise. You might also apply to the Central Bureau for the Employment of Women, 60 Chancery Lane, W.C.

*Consumptives.*

(113) Can you tell me where to write for information about consumptives at Davos? Can you recommend me a book giving information about Tasmania or any other part suitable for consumptives?—*S. G.*

You had much better consult a specialist in diseases of the chest. You would then know exactly which place would be most likely to benefit the patient on whose account you make inquiries.

*L.O.S.*

(114) Can you tell me if there is a resident medical officer at the East End Mothers' Home, as I wish very much to prepare for the L.O.S.? Is it necessary that there should be a resident medical officer?—*Nurse.*

No; there is no necessity. The training at the East End Mothers' Home is excellent.

**Useful Handbooks for Nurses.**

"Nurses' Dictionary of Medical Terms." Cloth, 2s.; leather, 2s. 6d.; post free 2s. 8d.

"On Preparation for Operation in Private Houses." 6d.

"Hospital Sisters and their Duties." 2s. 6d.

"Medical Gymnastics, including the Schott (Nauheim) Movements." 2s. 6d.

"The Human Body." 5s.

"Practical Handbook of Midwifery." 6s.

"A Handbook for Nurses." (Illustrated.) 5s.

"Tendencies to Consumption: How to Counteract Them." 2s. 6d.

"Syllabus of Lectures to Nurses." 1s.

The above works are published by the SCIENTIFIC PRESS, Ltd., and may be obtained through any bookseller or direct from the publisher, 28 and 29 Southampton Street, Strand, London, W.C.

